# DNA barcodes reveal microevolutionary signals in fire response trait in two legume genera

**DOI:** 10.1093/aobpla/plv124

**Published:** 2015-10-27

**Authors:** Abubakar Bello, Barnabas H. Daru, Charles H. Stirton, Samson B. M. Chimphango, Michelle van der Bank, Olivier Maurin, A. Muthama Muasya

**Affiliations:** 1 Bolus Herbarium, Biological Sciences Department, University of Cape Town, Private Bag X3, Rondebosch 7700, South Africa; 2 Department of Plant Science, University of Pretoria, Private Bag X20, Hatfield 0028, Pretoria, South Africa; 3 African Centre for DNA Barcoding, Department of Botany and Plant Biotechnology, University of Johannesburg, PO Box 524, Auckland Park 2006, Johannesburg, South Africa

**Keywords:** Fabaceae, *Otholobium*, *Psoralea*, reseeders, resprouters, South Africa

## Abstract

Large-scale DNA barcoding provides a new technique for species identification and evaluation of relationships across various levels (populations and species) and may reveal fundamental processes in recently diverged species. Here, we analysed DNA sequence variation in the recently diverged legumes from the Psoraleeae (Fabaceae) occurring in the Cape Floristic Region (CFR) of southern Africa to test the utility of DNA barcodes in species identification and discrimination. We further explored the phylogenetic signal on fire response trait (reseeding and resprouting) at species and generic levels. We showed that Psoraleoid legumes of the CFR exhibit a barcoding gap yielding the combination of *matK* and *rbcLa* (*matK* + *rbcLa*) data set as a better barcode than single regions. We found a high score (100 %) of correct identification of individuals to their respective genera but a very low score (<50 %) in identifying them to species. We found a considerable match (54 %) between genetic species and morphologically delimited species. We also found that different lineages showed a weak but significant phylogenetic conservatism in their response to fire as reseeders or resprouters, with more clustering of resprouters than would be expected by chance. These novel microevolutionary patterns might be acting continuously over time to produce multi-scale regularities of biodiversity. This study provides the first insight into the DNA barcoding campaign of land plants in species identification and detection of the phylogenetic signal in recently diverged lineages of the CFR.

## Abstract (precis)

This article is one of the series of publications carried out as part of the ongoing revision of the genus *Psoralea.* The genus was last revised in 1930 by Miss Helena Forbes. Since then, no thorough revision has been carried out on the genus beside the 1981 generic changes by Prof C.H. Stirton where a new genus *Otholobium* is described. The two genera represent a recent and rapid diversification of a lineage with a center of diversity and endemism in the Cape Floristic Region of South Africa.

## Introduction

The primary goal of DNA barcoding is the identification of an unknown sample by correctly matching a specific genetic marker to a reference sequence library. However, DNA barcoding can also be used as a tool for addressing fundamental questions in ecology, evolution and conservation biology (Kress *et al*. 2015). For evolutionary biologists and ecologists, one of the goals of DNA barcoding is to understand the origin of species and the factors causing the difference in species richness in different biomes across the globe. Generally, the full diversity of species in most diverse habitats is still poorly known (Kress *et al*. 2015). The primary focus of this article is to explore the application of DNA barcoding in some recently diverged lineages of an exceptionally unique fire derived biodiversity hotspot to determine its efficacy in species identification and detection of microevolutionary signals.

**Figure 1.**
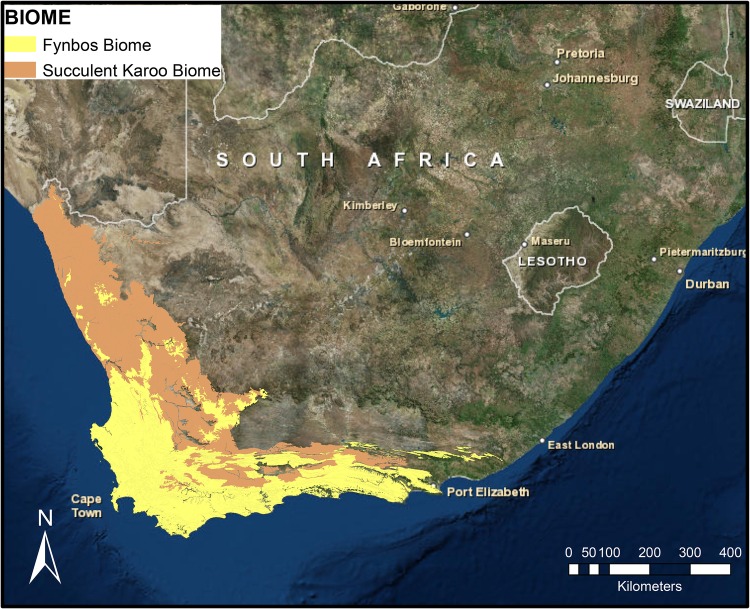
Map of the GCFR showing the Fynbos and the Succulent Karoo Biomes constructed based on Mucina and Rutherford (2006).

The Greater Cape Floristic Region (GCFR) is a home to Fynbos and the Succulent Karoo biomes—two major biodiversity hotspots located in the winter rainfall area of southern Africa (Myers *et al*. 2000) (Fig. 1). The Fynbos biome (also called the CFR) is famed for its high species diversity consisting of ∼9000 species of vascular plants packed into an area of 90 760 km^2^ of which ∼69 % are endemic (Manning and Goldblatt 2012). The family Fabaceae consists of ∼764 species in 43 genera. It is the second largest family in the CFR flora after Asteraceae. Three of the major clades of Fabaceae include the Crotalarieae (300 species), Podalyrieae (125 species) and African Psoraleeae (120 species). These legume lineages differ in their patterns of diversification, with Crotalarieae and Podalyrieae originating in the Eocene ca. 40 Ma (Edwards and Hawkins 2007; Schnitzler *et al*. 2011) and the African Psoraleeae originating during the Pliocene ca. 5 Ma (Egan and Crandall 2008). This suggests that the African Psoraleeae is a young lineage, which has undergone rapid recent radiation giving rise to ∼75 species of *Psoralea* L. and ∼53 species of *Otholobium* C.H.Stirt. (Stirton 2005; Manning and Goldblatt 2012). Majority of species in *Otholobium* and *Psoralea* have a narrow distribution and are frequently restricted to a single mountain stream or slope or a single soil type. In addition, several species are listed in the IUCN Red List under different levels of conservation categories ranging from extinct in the wild (e.g. *Psoralea gueinzii* and *P. cataracta*) to endangered (e.g. *Otholobium bowieanum*, *O. incanum*, *P. fascicularis* and *P. filifolia*) and vulnerable (*O. hamatum*, *O. venustum*, *P. abbottii* and *P. alata*) (Raimondo *et al*. 2009).

**Figure 2.**
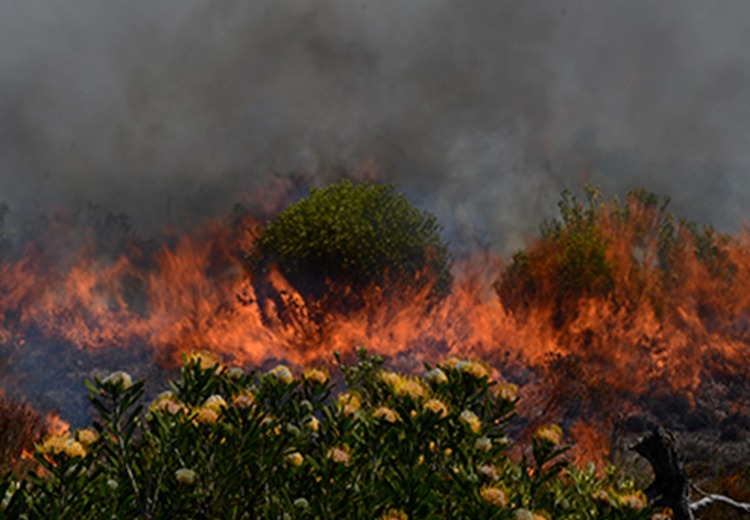
A recent fire burn in the Cape Fynbos, Table Mountain on 5 March 2015. Photograph: A.B.

Fynbos is a fire prone vegetation that requires regular burning for its persistence. The high species richness in the Fynbos biome has been ascribed to fire (Cowling *et al*. 1996; Linder 2003; Power *et al*. 2011). Plants adapt to fires in two major ways: as resprouters or reseeders (Bell 2001). Resprouting plants survive fire as individuals and then replace the lost structures by resprouting from surviving tissues. Conversely, reseeding individuals are often killed by fire (Fig. 2) and the population is re-established by a new generation growing from seeds (Bell 2001). Fire-survival and regeneration strategies of plants have been the subject of numerous studies (e.g. Keeley 1977; Bond 1985; Le Maitre and Midgley 1992; Schutte *et al*. 1995; Pausas and Keeley 2014; Scott *et al*. 2014). Cowling (1987) postulated that the high species diversity in the Gondwanan floras (Australian kwongan and Cape fynbos) may be ascribed to recurrent fires, edaphic specialization and short dispersal distance. There are noticeable differences in the allocation of resources to reserve storage, vegetative growth and reproductive effort linked with these fire-survival strategies (Bond and van Wilgen 1996; Bell 2001; Bond and Midgley 2001; Scott *et al*. 2014). For example, while reseeders are generally characterized by a shorter lifespan, they tend to grow rapidly and taller with much allocation of resources predominantly above ground. Resprouters, on the other hand, have longer lifespans, slower growth, produce fewer seeds and have a below ground resource allocation in starch-rich lignotubers (Hansen *et al*. 1991; Bell and Ojeda 1999). Reseeders produce larger numbers of viable seeds than do resprouters due to their greater reliance on seed for survival (Hansen *et al*. 1991; Bell 2001), resulting in elevated post-fire recruitment. There are also reported differences in seed yield and quality with reseeders having higher N and P concentrations in the seeds than those of congeneric resprouters (Hansen *et al*. 1991). Other differences include nutritional requirements with reseeders requiring more nutrients than the resprouters due to the high nutritional costs of seed production and growth (Hansen *et al*. 1991; Bell 2001). These strategies influence speciation rates in woody genera in the fynbos (Wells 1969; Litsios *et al*. 2014), with reseeders shown to have higher diversification rates than resprouters (Litsios *et al*. 2014). Other studies have shown that fire-survival and regeneration strategy (reseeding/resprouting) is a character of taxonomic, ecological and evolutionary importance in Fynbos legumes (Schutte *et al*. 1995; Litsios *et al*. 2014; Scott *et al*. 2014).

**Figure 3.**
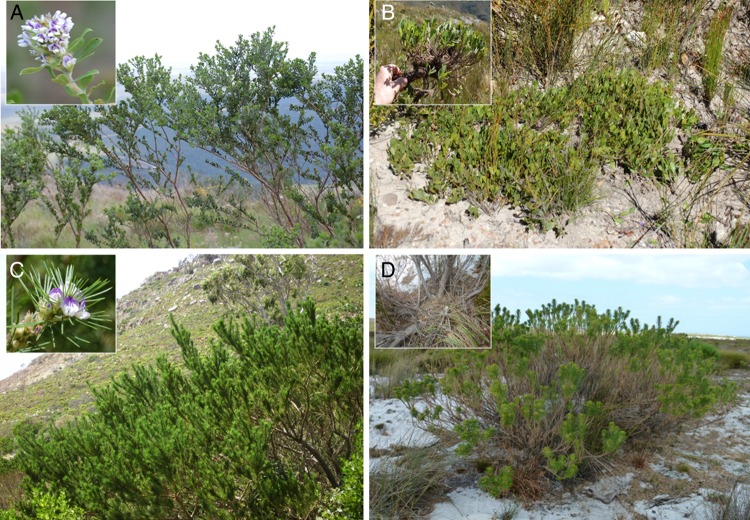
Habit in *Otholobium* and *Psoralea* species: (A) reseeding, *O. spicatum*; (B) resprouting, *O. rotundifolium*; (C) reseeding, *P. pinnata*; (D) resprouting, *Psoralea* sp. nov. Photographs: C.H.S. (A–C) and A.B. (D).

Traditionally, species identification depends primarily on morphological features (morphospecies). As molecular data became increasingly available and new techniques such as DNA barcoding emerged, species identification is becoming fast, reliable and more accurate. Here, we use *matK* and *rbcLa* and the combination of the two regions (*matK* + *rbcLa*), based on their recognition as core plant barcode markers by the Consortium for the Barcode of Life Plant Working Group (CBOL 2009) to (i) test their efficacy in identifying species of two southern African Psoraleoid genera (*Otholobium* and *Psoralea*); (ii) explore the potential of the DNA barcode markers in grouping Psoraleoid legume sequences into molecular operational taxonomic units (MOTUs) or genetic species units and (iii) test the power of DNA barcodes in revealing microevolutionary patterns including fire-survival and regeneration strategies. The genera *Otholobium* and *Psoralea* were chosen for this study because they both have species with reseeding and resprouting modes of regeneration (Fig. 3). Furthermore, although the two genera are closely related (Dludlu *et al*. 2013), they differ markedly in terms of their morphology and ecology. For example, *Otholobium* species differ from *Psoralea* by the absence of a cupulum on the flower pedicel (unique structure in *Psoralea*, Tucker and Stirton 1991); possession of entire recurved mucronate-obovate to oblanceolate leaflets and inflorescences characterized by bracteate triplets of flowers, with each triplet subtended by a single variously shaped bract (Stirton 1981). Leaves of *Psoralea* range from 1- to 19-foliolate compound structures or reduced to small-scale-like structures with only *P. aculeata* having a recurved mucro (Stirton 1989; Manning and Goldblatt 2012), and each flower is subtended by a pair of free minute bracts. The two genera also differ in terms of habitat preferences. Eighty per cent of *Psoralea* species inhabit seeps, marshes, riverbanks and/or moist, mist laden high-altitude habitats, while *Otholobium* species occur predominantly in drier habitats, with only 11 % of species occupying the moist habitats favoured by *Psoralea* (Stirton 1989; Manning and Goldblatt 2012).

## Methods

### Taxon sampling

We collected 172 samples representing 26 species of *Otholobium* and 43 species of *Psoralea* across their distribution range in the CFR. Where possible, each species was represented by two or more different samples. In all, we collected 72 samples of *Otholobium* and 100 samples of *Psoralea* (voucher specimens are deposited at the Bolus Herbarium (BOL) and listed in Table 1). Of these samples, 23 out of the 26 species of *Otholobium* and 26 out of 43 species of *Psoralea* are represented by more than one sample. Only samples for which sequences for both genes (*matK* and *rbcLa*) are available were included in the analyses. The final data set used in the analyses included 4 reseeding (27 samples) and 22 resprouting (35 samples) species of *Otholobium*, and 35 (43 samples) reseeding and 8 (56 samples) resprouting species of *Psoralea*. Information on fire response strategy was extracted from Stirton (1989), Manning and Goldblatt (2012) and Snijman (2013). To our knowledge, no species included in our analysis show both fire response strategies in wild populations. Collection details including GPS coordinates, altitude and photographs of specimens are available online in the Barcode of Life Data Systems (BOLD; www.boldsystems.org) together with DNA sequences.

**Table 1.** List of voucher specimens and the DNA sequence BOLD ID reference number.

Taxon name	Collector	Number	BOLD ID	Herbarium	Distribution
*Otholobium acuminatum*	Muasya & Stirton	AMM3850	FAUCT199-11	BOL	Africa
*Otholobium acuminatum*	Muasya & Stirton	AMM3603	FAUCT144-11	BOL	Africa
*Otholobium arborescens*	Muasya & Stirton	AMM3279	FAUCT051-11	BOL	Africa
*Otholobium beanianum* sp. nov*.*	Muasya & Stirton	AMM3350	FAUCT067-11	BOL	Africa
*Otholobium bracteolatum*	Muasya & Stirton	AMM3963	FAUCT229-11	BOL	Africa
*Otholobium bracteolatum*	Muasya & Stirton	AMM3164	FAUCT002-11	BOL	Africa
*Otholobium bracteolatum*	Muasya & Stirton	AMM3879	FAUCT208-11	BOL	Africa
*Otholobium bracteolatum* ssp. *limnophilum* ssp. nov*.*	Muasya & Stirton	AMM & Stirton 13155	FAUCT367-11	BOL	Africa
*Otholobium bracteolatum* ssp. *limnophilum* ssp. nov*.*	Muasya & Stirton	AMM3204	FAUCT030-11	BOL	Africa
*Otholobium candicans*	Muasya & Stirton	AMM3911	FAUCT223-11	BOL	Africa
*Otholobium candicans*	Muasya & Stirton	AMM3369	FAUCT072-11	BOL	Africa
*Otholobium candicans*	Muasya & Stirton	AMM3563	FAUCT130-11	BOL	Africa
*Otholobium crewii* sp. nov*.*	Muasya & Stirton	AMM3264	FAUCT041-11	BOL	Africa
*Otholobium flexuosum*	Muasya & Stirton	AMM3276	FAUCT049-11	BOL	Africa
*Otholobium flexuosum*	Muasya & Stirton	AMM3280	FAUCT052-11	BOL	Africa
*Otholobium fruticans*	Muasya & Stirton	AMM3480	FAUCT106-11	BOL	Africa
*Otholobium fruticans*	Muasya & Stirton	AMM3397	FAUCT081-11	BOL	Africa
*Otholobium hamatum*	Muasya & Stirton	AMM3310	FAUCT060-11	BOL	Africa
*Otholobium hamatum*	Muasya & Stirton	AMM3306	FAUCT059-11	BOL	Africa
*Otholobium hirtum*	Muasya & Stirton	AMM3326	FAUCT063-11	BOL	Africa
*Otholobium hirtum*	Muasya & Stirton	AMM3991	FAUCT232-11	BOL	Africa
*Otholobium hirtum*	Muasya & Stirton	AMM3190	FAUCT018-11	BOL	Africa
*Otholobium hirtum*	Muasya & Stirton	AMM3373	FAUCT074-11	BOL	Africa
*Otholobium hirtum*	Muasya & Stirton	AMM3372	FAUCT073-11	BOL	Africa
*Otholobium hirtum*	Muasya & Stirton	AMM3499	FAUCT112-11	BOL	Africa
*Otholobium hirtum*	Muasya & Stirton	AMM3878	FAUCT207-11	BOL	Africa
*Otholobium lucens* sp. nov*.*	Muasya & Stirton	AMM3570	FAUCT133-11	BOL	Africa
*Otholobium mundianum*	Muasya & Stirton	AMM3885	FAUCT211-11	BOL	Africa
*Otholobium obliquum*	Muasya & Stirton	AMM3198.1	FAUCT023-11	BOL	Africa
*Otholobium parviflorum*	Muasya & Stirton	AMM3199	FAUCT024-11	BOL	Africa
*Otholobium parviflorum*	Muasya & Stirton	AMM3542	FAUCT119-11	BOL	Africa
*Otholobium prodiens*	Muasya & Stirton	AMM3845	FAUCT196-11	BOL	Africa
*Otholobium prodiens*	Muasya & Stirton	AMM3854	FAUCT201-11	BOL	Africa
*Otholobium pustulatum*	Muasya & Stirton	AMM3286	FAUCT054-11	BOL	Africa
*Otholobium rotundifolium*	Muasya & Stirton	AMM3929	FAUCT227-11	BOL	Africa
*Otholobium rotundifolium*	Muasya & Stirton	AMM3173	FAUCT009-11	BOL	Africa
*Otholobium rubicundum*	Muasya & Stirton	AMM5982	FAUCT359-11	BOL	Africa
*Otholobium schutteae* sp. nov*.*	Muasya & Stirton	AMM3575	FAUCT134-11	BOL	Africa
*Otholobium spicatum*	Muasya & Stirton	AMM3445	FAUCT097-11	BOL	Africa
*Otholobium spicatum*	Muasya & Stirton	AMM3498	FAUCT111-11	BOL	Africa
*Otholobium spicatum*	Muasya & Stirton	AMM3906	FAUCT220-11	BOL	Africa
*Otholobium spicatum*	Muasya & Stirton	AMM3568	FAUCT132-11	BOL	Africa
*Otholobium stachyerum*	Muasya & Stirton	AMM3837	FAUCT194-11	BOL	Africa
*Otholobium stachyerum*	Muasya & Stirton	AMM3872	FAUCT206-11	BOL	Africa
*Otholobium stachyerum*	Muasya & Stirton	AMM3791	FAUCT183-11	BOL	Africa
*Otholobium stachyerum*	Muasya & Stirton	AMM3604	FAUCT145-11	BOL	Africa
*Otholobium stachyerum*	Muasya & Stirton	AMM3851	FAUCT200-11	BOL	Africa
*Otholobium striatum*	Muasya & Stirton	AMM3339	FAUCT064-11	BOL	Africa
*Otholobium striatum*	Muasya & Stirton	AMM3363	FAUCT071-11	BOL	Africa
*Otholobium striatum*	Muasya & Stirton	AMM3561	FAUCT129-11	BOL	Africa
*Otholobium striatum*	Muasya & Stirton	AMM4106	FAUCT247-11	BOL	Africa
*Otholobium striatum*	Muasya & Stirton	AMM3351	FAUCT068-11	BOL	Africa
*Otholobium striatum*	Muasya & Stirton	AMM3318	FAUCT062-11	BOL	Africa
*Otholobium thomii*	Muasya & Stirton	AMM3187	FAUCT016-11	BOL	Africa
*Otholobium uncinatum*	Muasya & Stirton	AMM3175	FAUCT010-11	BOL	Africa
*Otholobium uncinatum*	Muasya & Stirton	AMM3263	FAUCT040-11	BOL	Africa
*Otholobium uncinatum*	Muasya & Stirton	AMM3261	FAUCT038-11	BOL	Africa
*Otholobium velutinum*	Muasya & Stirton	AMM & Stirton 13106	FAUCT362-11	BOL	Africa
*Otholobium virgatum*	Muasya & Stirton	AMM3908	FAUCT222-11	BOL	Africa
*Otholobium virgatum*	Muasya & Stirton	AMM3395	FAUCT079-11	BOL	Africa
*Otholobium virgatum*	Muasya & Stirton	AMM3163	FAUCT001-11	BOL	Africa
*Otholobium virgatum*	Muasya & Stirton	AMM3191	FAUCT019-11	BOL	Africa
*Psoralea aculeata*	Muasya & Stirton	AMM3183	FAUCT012-11	BOL	Africa
*Psoralea aculeata*	Muasya & Stirton	AMM3405	FAUCT088-11	BOL	Africa
*Psoralea aculeata*	Muasya & Stirton	AMM3550	FAUCT124-11	BOL	Africa
*Psoralea aculeata*	Muasya & Stirton	AMM3170	FAUCT006-11	BOL	Africa
*Psoralea affinis*	Muasya & Stirton	AMM3903.2	FAUCT215-11	BOL	Africa
*Psoralea affinis*	Muasya & Stirton	AMM3868	FAUCT203-11	BOL	Africa
*Psoralea alata*	Muasya & Stirton	AMM3262	FAUCT039-11	BOL	Africa
*Psoralea alata*	Muasya & Stirton	AMM3398	FAUCT082-11	BOL	Africa
*Psoralea alata*	Muasya & Stirton	AMM3880	FAUCT209-11	BOL	Africa
*Psoralea alata*	Muasya & Stirton	AMM3901	FAUCT213-11	BOL	Africa
*Psoralea aphylla*	Muasya & Stirton	AMM3400	FAUCT084-11	BOL	Africa
*Psoralea arborea*	Muasya & Stirton	AMM3212	FAUCT032-11	BOL	Africa
*Psoralea arborea*	Muasya & Stirton	AMM3248	FAUCT037-11	BOL	Africa
*Psoralea arida* sp. nov*.*	Muasya & Stirton	AMM3526	FAUCT113-11	BOL	Africa
*Psoralea arida* sp. nov*.*	Muasya & Stirton	AMM4098	FAUCT246-11	BOL	Africa
*Psoralea asarina*	Muasya & Stirton	AMM3907	FAUCT221-11	BOL	Africa
*Psoralea asarina*	Muasya & Stirton	AMM3476	FAUCT105-11	BOL	Africa
*Psoralea asarina*	Muasya & Stirton	AMM3552	FAUCT126-11	BOL	Africa
*Psoralea axillaris*	Muasya & Stirton	AMM3834	FAUCT192-11	BOL	Africa
*Psoralea axillaris*	Muasya & Stirton	AMM3848	FAUCT198-11	BOL	Africa
*Psoralea axillaris*	Muasya & Stirton	AMM3827	FAUCT191-11	BOL	Africa
*Psoralea axillaris*	Muasya & Stirton	AMM5874	FAUCT356-12	BOL	Africa
*Psoralea brilliantissima* sp. nov*.*	Muasya & Stirton	AMM3621	FAUCT152-11	BOL	Africa
*Psoralea* cf. *latifolia*	Muasya & Stirton	AMM4028	FAUCT234-11	BOL	Africa
*Psoralea congesta*	Muasya & Stirton	AMM5462	FAUCT328-11	BOL	Africa
*Psoralea elegans* sp. nov.	Muasya & Stirton	AMM3591	FAUCT139-11	BOL	Africa
*Psoralea filifolia*	Muasya & Stirton	AMM4321	FAUCT278-11	BOL	Africa
*Psoralea fleta*	Muasya & Stirton	AMM3273	FAUCT047-11	BOL	Africa
*Psoralea fleta*	Muasya & Stirton	AMM3341	FAUCT065-11	BOL	Africa
*Psoralea fleta*	Muasya & Stirton	AMM3342	FAUCT066-11	BOL	Africa
*Psoralea forbesii* sp. nov*.*	Muasya & Stirton	AMM3578	FAUCT135-11	BOL	Africa
*Psoralea forbesii* sp. nov*.*	Muasya & Stirton	AMM3592	FAUCT140-11	BOL	Africa
*Psoralea gigantea*	Muasya & Stirton	AMM3203	FAUCT029-11	BOL	Africa
*Psoralea glaucescens*	Muasya & Stirton	AMM3289	FAUCT056-11	BOL	Africa
*Psoralea glaucescens*	Muasya & Stirton	AMM3312	FAUCT061-11	BOL	Africa
*Psoralea imbricata*	Muasya & Stirton	AMM4030	FAUCT235-11	BOL	Africa
*Psoralea imbricata*	Muasya & Stirton	AMM3439	FAUCT094-11	BOL	Africa
*Psoralea imbricata*	Muasya & Stirton	AMM3544	FAUCT120-11	BOL	Africa
*Psoralea imbricata*	Muasya & Stirton	AMM3904	FAUCT218-11	BOL	Africa
*Psoralea imbricata*	Muasya & Stirton	AMM3396	FAUCT080-11	BOL	Africa
*Psoralea imbricata*	Muasya & Stirton	AMM3399	FAUCT083-11	BOL	Africa
*Psoralea imminens* sp. nov.	Muasya & Stirton	AMM3596	FAUCT141-11	BOL	Africa
*Psoralea ivumba* sp. nov*.*	Muasya & Stirton	AMM3374	FAUCT075-11	BOL	Africa
*Psoralea ivumba* sp. nov*.*	Muasya & Stirton	AMM3165	FAUCT003-11	BOL	Africa
*Psoralea keetii*	Muasya & Stirton	AMM3599	FAUCT143-11	BOL	Africa
*Psoralea laevigata*	Muasya & Stirton	AMM3457	FAUCT099-11	BOL	Africa
*Psoralea laxa*	Muasya & Stirton	AMM3646	FAUCT156-11	BOL	Africa
*Psoralea laxa*	Muasya & Stirton	AMM4325	FAUCT279-11	BOL	Africa
*Psoralea laxa*	Muasya & Stirton	AMM3548	FAUCT122-11	BOL	Africa
*Psoralea laxa*	Muasya & Stirton	AMM3870	FAUCT205-11	BOL	Africa
*Psoralea muirii* sp. nov*.*	Muasya & Stirton	AMM4181	FAUCT257-11	BOL	Africa
*Psoralea odoratissima*	Muasya & Stirton	AMM3532	FAUCT116-11	BOL	Africa
*Psoralea odoratissima*	Muasya & Stirton	AMM3557	FAUCT127-11	BOL	Africa
*Psoralea oligophylla*	Muasya & Stirton	AMM3798	FAUCT185-11	BOL	Africa
*Psoralea oreophila*	Muasya & Stirton	AMM3463	FAUCT102-11	BOL	Africa
*Psoralea oreophila*	Muasya & Stirton	AMM3464	FAUCT103-11	BOL	Africa
*Psoralea oreopola* sp. nov*.*	Muasya & Stirton	AMM4370	FAUCT283-11	BOL	Africa
*Psoralea oreopola* sp. nov*.*	Muasya & Stirton	AMM4376	FAUCT285-11	BOL	Africa
*Psoralea oreopola* sp. nov*.*	Muasya & Stirton	AMM3271	FAUCT044-11	BOL	Africa
*Psoralea pinnata*	Muasya & Stirton	AMM3169	FAUCT005-11	BOL	Africa
*Psoralea pinnata*	Muasya & Stirton	AMM3403	FAUCT086-11	BOL	Africa
*Psoralea pinnata*	Muasya & Stirton	AMM3186	FAUCT015-11	BOL	Africa
*Psoralea pinnata*	Muasya & Stirton	AMM3547	FAUCT121-11	BOL	Africa
*Psoralea pinnata*	Muasya & Stirton	AMM3172	FAUCT008-11	BOL	Africa
*Psoralea pinnata*	Muasya & Stirton	AMM3171	FAUCT007-11	BOL	Africa
*Psoralea pinnata*	Muasya & Stirton	AMM3189	FAUCT017-11	BOL	Africa
*Psoralea plauta*	Muasya & Stirton	AMM3611	FAUCT149-11	BOL	Africa
*Psoralea pullata*	Muasya & Stirton	AMM3178	FAUCT011-11	BOL	Africa
*Psoralea pullata*	Muasya & Stirton	AMM3903.1	FAUCT214-11	BOL	Africa
*Psoralea repens*	Muasya & Stirton	AMM3809	FAUCT186-11	BOL	Africa
*Psoralea repens*	Muasya & Stirton	AMM3168	FAUCT004-11	BOL	Africa
*Psoralea restioides*	Muasya & Stirton	AMM3216	FAUCT033-11	BOL	Africa
*Psoralea rhizotoma* sp. nov.	Muasya & Stirton	AMM3659	FAUCT158-11	BOL	Africa
*Psoralea rigidula*	Muasya & Stirton	AMM3390	FAUCT077-11	BOL	Africa
*Psoralea sordida* sp. nov.	Muasya & Stirton	AMM3579	FAUCT136-11	BOL	Africa
*Psoralea sordida* sp. nov.	Muasya & Stirton	AMM3580	FAUCT137-11	BOL	Africa
*Psoralea sparsa* sp. nov*.*	Muasya & Stirton	AMM3567	FAUCT131-11	BOL	Africa
*Psoralea speciosa*	Muasya & Stirton	AMM3458	FAUCT100-11	BOL	Africa
*Psoralea speciosa*	Muasya & Stirton	AMM3610	FAUCT148-11	BOL	Africa
*Psoralea speciosa*	Muasya & Stirton	AMM3456	FAUCT098-11	BOL	Africa
*Psoralea speciosa*	Muasya & Stirton	AMM3607	FAUCT146-11	BOL	Africa
*Psoralea suaveolens* sp. nov.	Muasya & Stirton	AMM4396	FAUCT286-11	BOL	Africa
*Psoralea suaveolens* sp. nov.	Muasya & Stirton	AMM4975	FAUCT303-11	BOL	Africa
*Psoralea triflora* sp. nov.	Muasya & Stirton	AMM3862	FAUCT202-11	BOL	Africa
*Psoralea usitata*	Muasya & Stirton	AMM4344	FAUCT281-11	BOL	Africa
*Psoralea usitata*	Muasya & Stirton	AMM4071	FAUCT244-11	BOL	Africa
*Psoralea usitata*	Muasya & Stirton	AMM3440	FAUCT095-11	BOL	Africa
*Psoralea usitata*	Muasya & Stirton	AMM3528	FAUCT114-11	BOL	Africa
*Psoralea usitata*	Muasya & Stirton	AMM3541	FAUCT118-11	BOL	Africa
*Psoralea usitata*	Muasya & Stirton	AMM3194	FAUCT020-11	BOL	Africa
*Psoralea usitata*	Muasya & Stirton	AMM3414	FAUCT092-11	BOL	Africa
*Psoralea usitata vigilans* sp. nov*.*	Muasya & Stirton	AMM3415	FAUCT093-11	BOL	Africa
*Psoralea usitata vigilans* sp. nov*.*	Muasya & Stirton	AMM4340	FAUCT280-11	BOL	Africa
*Psoralea verrucosa*	Muasya & Stirton	AMM3357	FAUCT070-11	BOL	Africa
*Psoralea verrucosa*	Muasya & Stirton	AMM3905	FAUCT219-11	BOL	Africa
*Psoralea verrucosa*	Muasya & Stirton	AMM3353	FAUCT069-11	BOL	Africa
*Psoralea verrucosa*	Muasya & Stirton	AMM3269	FAUCT042-11	BOL	Africa
*Psoralea verrucosa*	Muasya & Stirton	AMM4371	FAUCT284-11	BOL	Africa
*Psoralea verrucosa*	Muasya & Stirton	AMM3270	FAUCT043-11	BOL	Africa

### DNA extraction, sequencing and alignment

All the samples were sent to the Canadian Centre for DNA Barcoding (CCDB) in Canada, where total DNA was extracted and the two core DNA barcodes (*matK* and *rbcLa*) were sequenced according to standard CCDB protocols (Ivanova *et al*. 2006). Sequence alignment was performed using Multiple Sequence Comparison by Log Expectation (MUSCLE v. 3.8.31, Edgar 2004) plugin in Geneious v.8.0.4 (Kearse *et al*. 2012) and manually adjusted using MESQUITE v.2.5 (Maddison and Maddison 2008). The two regions were aligned separately and then combined.

### Evaluation of DNA barcodes

First, we evaluated the performance of the DNA markers (*matK*, *rbcLa* and *matK* + *rbcLa*) in species identification and delimitation of African Psoraleoid legumes at species and generic levels by applying two criteria commonly used to evaluate the utility of the DNA barcodes in species discrimination: the barcode gap of Meyer and Paulay (2005) and discriminatory power (Hebert *et al*. 2004*b*). Barcode gap was assessed by comparing intraspecific variation (i.e. the amount of genetic variation within species) to interspecific variation (between species). A good barcode should exhibit a significant gap, meaning that sequence variation within species should be significantly lower than between species. Statistical significance between intra- and interspecific variation was assessed using Wilcoxon test in R (R Core Team 2013).

The discriminatory power of DNA barcoding was tested by evaluating the proportion of correct species identification at different taxonomic level (species and generic) using *matK*, *rbcLa* and *matK* + *rbcLa* regions. All sequences were labelled according to the names of the species from which the sequences were generated. The test of discriminatory power was carried out using two methods: the ‘best close match’ (Meier *et al*. 2006) and the ‘near neighbour’ using the functions bestCloseMatch and nearNeighbour implemented in the R package Spider (Brown *et al*. 2012). Before the test, we determined the optimized genetic distance suitable as threshold for taxon identification using the function localMinima also implemented in Spider (Brown *et al*. 2012).

The function bestCloseMatch conducts the ‘best close match’ analysis (Meier *et al*. 2006) by searching for the closest individual in the data set. If the closest individual is within a given threshold, the outcome is scored as ‘correct’, and if it is further, then the result is ‘no ID’ (no identification). If more than one species is tied for closest match, the outcome of the test is an ‘ambiguous’ identification. When all matches within the threshold are different species to the query, the result is scored as ‘incorrect’. The nearNeighbour function finds the closest individual and returns the score ‘true’ (similar to ‘correct’ in the bestCloseMatch method) if their names are the same, but if the names are different, the outcome is scored as ‘false’ (similar to ‘incorrect’ in the bestCloseMatch method).

### Barcode test of species delimitation

Apart from investigating the potential of DNA markers in identifying species, we explored their ability in assigning morphologically delimited species into genetic units, i.e. ‘MOTUs’ or ‘genetic species’ (*sensu* Saunders and McDevit 2013). We considered MOTUs as groupings or clusters of specimens that fall around a medoid. The goal is to verify the optimal number of clusters (species) that may be inferred from the pairwise genetic distance matrices of Psoraleoid legumes. A match between our genetic species and morphologically delimited species would indicate that one could serve as a surrogate for the other (see Stahlhut *et al*. 2013), and thus lend support to the discriminatory power of DNA barcoding. We used partition around medoids (PAM) approach using the R package Cluster (Mächler *et al*. 2015; R Core Team 2015). Our decision in choosing PAM was made after testing the performance of several clustering algorithms including ‘Agglomerative Nesting (agnes)’, ‘Divisive Analysis Clustering (diana)’ and ‘Fuzzy Analysis Clustering (fanny)’. Results from these other approaches were not reported for at least one of the two main reasons. Firstly, they yielded identical results to PAM and are less straight forward to explain. For example, fanny does not produce unique clusters. Instead, it groups each species (probabilistically) to multiple clusters. The second reason was that the methodologies employed by some of the algorithms do not easily accommodate the restriction of cluster sizes.

The PAM algorithm works as follows: given a specific number of clusters (*k*), desired from a distance matrix, PAM searches for species (here referred to as medoids) that are representative of the data. The number of medoids sought is usually the same as the number of desired clusters *k*. Each cluster is then constructed such that the distance of any other sample, in the cluster, from its medoid is minimal. Cluster sizes between 2 and 69 were first investigated for each distance matrix. An optimal cluster size was then chosen as the one that yielded the maximum silhouette coefficient (Kaufman and Rousseeuw 1990). A silhouette coefficient measures the quality of clustering, derived as an average of the silhouette widths over all species. We used the silhouette width as an aggregate of a measure of the suitability of a cluster for each observation it contains relative to the next best cluster for the observations. Silhouette coefficients range between 0 and 1.

### Barcode test for phylogenetic signal

We explored the potential of the DNA barcode data to reveal microevolutionary patterns by testing for phylogenetic signal in the affinity of lineages to fire-survival and regeneration strategies. We used a phylogeny of the southern African Psoraleoid species and a binary matrix of reseeders versus resprouters. The phylogeny was reconstructed using a combination of *matK* and *rbcLa* data sets, based on a maximum-likelihood (ML) approach (Stamatakis *et al*. 2008), enforcing topological constraints from a consensus tree of the Bayesian analysis of the data set. We used the GTR + G + I substitution model based on the result of Akaike information criterion from Modeltest v.2.3 (Nylander 2004), and ran 1000 ML searches. Phylogenetic signal was tested on the ML best tree and binary matrix of reseeders versus resprouters using the *D* statistics of Fritz and Purvis (2010) in the R package Caper (Orme *et al*. 2012). The *D* statistics calculates the sum of changes of a binary trait along the branches of a phylogeny, and compares it with a random model and clumping expected under a Brownian evolution. Significance was assessed by shuffling the trait values 999 times at the tips of the phylogeny. *D* = 1 corresponds to a random distribution of traits at the tip of the phylogeny; *D* = 0 corresponds to a Brownian motion model (Fritz and Purvis 2010).

## Results

**Figure 4.**
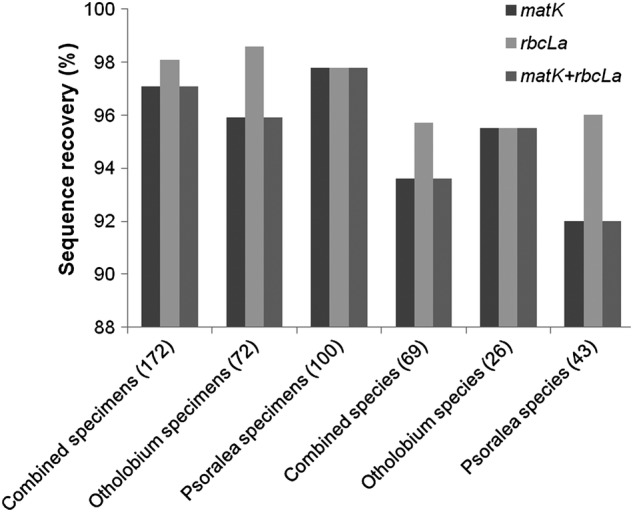
Percentage of specimens and species of *Otholobium* and *Psoralea* from which *rbcL* and *matK* barcodes were recovered. Numbers in parentheses are the total number of individuals (specimens, species).

For the core barcode loci, we obtained 332 sequences (165 and 167 for *matK* and *rbcLa*, respectively) from 172 specimens representing 72 *Otholobium* and 100 *Psoralea*. Sequence recoverability was higher for *rbcLa* than for *matK* (98.1 and 97.1 % of specimens, respectively, Fig. 4). The combined *matK* + *rbcLa* sequence data were obtained from 98.1 % of the specimens sampled (Fig. 4). For *rbcLa*, we recovered 95.7 % of the 69 species sequenced, and 93.6 % for *matK* and when combined with *rbcLa*, i.e. *matK* + *rbcLa*. Both barcodes combined yielded a total of 1326 bp (770 bp for *matK* and 549 bp for *rbcLa*).

**Figure 5.**
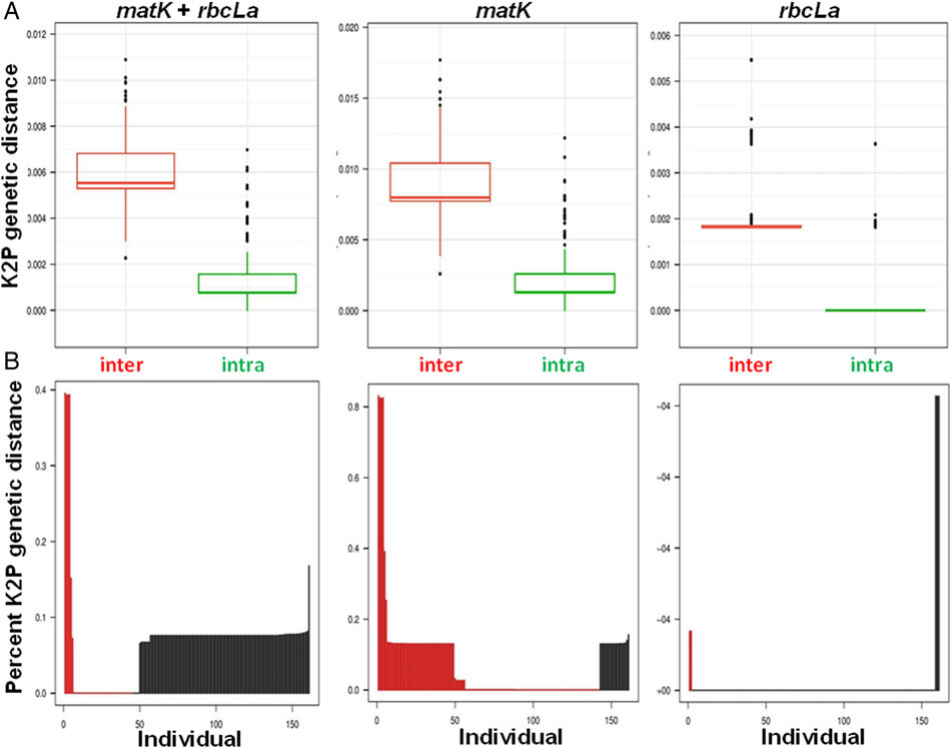
(A) Evaluation of barcode gap in the data set. Boxplot of the interspecific (inter) and intraspecific (intra) genetic distances for *matK* + *rbcLa*, *matK* and *rbcLa* data sets, indicating the existence of a barcode gap, i.e. minimum interspecific distance is greater than the maximum intraspecific distance. The bottom and top of the boxes show the first and third quartiles, respectively, the median is indicated by the horizontal line, the range of the data by the vertical line and outliers by dots. (B) Line plot of the barcode gap for the 171 Psoraleiod individuals. The black lines indicate where the minimum interspecific distance is greater than the maximum intraspecific distance (an indication of a barcode gap); the red lines show where this pattern is reversed, i.e. the situation where there is no barcoding gap.

The mean interspecific distances for the single and combined regions are lower than 1 %, ranging from 0.002013 in *rbcLa* to 0.008612 in *matK*. The mean intraspecific variation for each and combined DNA regions was also low, ranging from 0.000108 in *rbcLa* to 0.001251 in the combined data set, *matK* + *rbcLa*. The mean intraspecific distances in all cases are significantly lower than interspecific distances (Wilcoxon test, *P* < 0.0001). The minimum interspecific genetic distance is greater than the maximum intraspecific genetic distance in *matK* + *rbcLa* data set (Fig. 5A), indicating the existence of a barcode gap in the data set. The comparison between the lowest interspecific distances (red lines) versus the maximum intraspecific distances (black lines) is shown in Fig. 5B. Further, we found 72 % (116) of the individuals with barcode gap and 28 % (45) without a barcode gap in *matK* + *rbcLa* data set. We also found 12 % (19) of the individuals with barcode gap and 88 % (152) without a barcode gap in *matK* data set. Lastly, we found only 3 % (2) of the individuals with barcode gap in *rbcLa* data set and 97 % (168) without a barcode gap.

Testing the efficacy of DNA barcoding based on discriminatory potential shows that the calculated thresholds ranged from 0.045 in *matK* to an optimized value of 0.36 for the full data set (*matK* + *rbcLa*). Using these cut-offs, we found 100 % true and correct identification in all the data sets for the near-neighbour and best close match analyses, respectively, in identifying the individuals to their respective genera (*Psoralea* or *Otholobium*). In terms of identifying the individuals at the species level, we found 25 % success rate for *matK* compared with 4 % in *rbcLa* for the near-neighbour method, which did not improve when the two barcodes were combined (*matK* + *rbcLa*) (Table 2). Similarly, for the best close match analysis, *matK* + *rbcLa* and *matK* exhibited 11 % correct identification rate as opposed to failure in *rbcLa* (0 %) data set (Table 2).

**Table 2.** Performance of the DNA barcodes in identification of individuals to species or genera of Psoraleoid legumes evaluated based on discriminatory potential. Values in parenthesis represent identification of individuals to genera. ‘True’ indicates instances when the near-neighbour method finds the closest individual in the data set and their names are the same or ‘False’ if different. ‘Correct’, ‘Incorrect’, ‘Ambiguous’ and ‘No id’ are used in the best close match method, when the name of the closest match is the same, different, more than one species is the closest match and no species are within the threshold distance, respectively.

DNA barcoding regions	Number of genetic species (MOTUs)	Near neighbour		Best close match			
		True (%)	False (%)	Ambiguous (%)	Correct (%)	Incorrect (%)	No ID (%)
*matK* + *rbcLa*	36	25 (100)	75 (0)	51 (0)	11 (100)	38 (0)	0
*matK*	33	25 (100)	75 (0)	53 (0)	11 (100)	36 (0)	0
*rbcLa*	7	4 (100)	96 (0)	79 (0)	0 (100)	21 (0)	0

Of the 69 morphologically delimited species included in the analyses, varying discriminatory power in the performance of the DNA markers in grouping specimens into genetic species (MOTUs) was found. *rbcLa* grouped all the specimens into 7 genetic species only (silhouette coefficient = 0.98), followed by *matK* (33 genetic species; silhouette coefficient = 0.84; Table 3). The combination of *matK* + *rbcLa* grouped specimens into 37 genetic species unit (silhouette coefficient = 0.84). We, therefore, discussed our results based on the core barcode, i.e. *matK* + *rbcLa* data set.

**Table 3.** Genetic species delimited using the best DNA barcode region (*matK* + *rbcLa*) identified in this study.

No.	Composition of genetic species or MOTUs	
1	[1] *O. acuminatum* Muasya & Stirton3603	[15] *O. spicatum* Muasya & Stirton3906
	[2] *O. acuminatum* Muasya & Stirton3850	[16] *O. stachyerum* Muasya & Stirton3604
	[3] *O. arborescens* Muasya & Stirton3279	[17] *O. stachyerum* Muasya & Stirton3851
	[4] *O. candicans* Muasya & Stirton3369	[18] *O. stachyerum* Muasya & Stirton3872
	[5] *O. flexuosum* Muasya & Stirton3276	[19] *O. striatum* Muasya & Stirton3318
	[6] *O. flexuosum* Muasya & Stirton3280*.*1	[20] *O. striatum* Muasya & Stirton3339
	[7] *O. hirtum* Muasya & Stirton3499	[21] *O. striatum* Muasya & Stirton3351
	[8] *O. obliquum* Muasya & Stirton3198*.*1	[22] *O. striatum* Muasya & Stirton3363
	[9] *O. parviflorum* Muasya & Stirton3199	[23] *O. striatum* Muasya & Stirton3561
	[10] *O. pustulatum* Muasya & Stirton3286	[24] *O. striatum* Muasya & Stirton4106
	[11] *O. rotundifolium* Muasya & Stirton3173	[25] *O. thomii* Muasya & Stirton3187
	[12] *O. rotundifolium* Muasya & Stirton3929	[26] *O. uncinatum* Muasya & Stirton3261
	[13] *O. spicatum* Muasya & Stirton3498	[27] *O. uncinatum* Muasya & Stirton3263
	[14] *O. spicatum* Muasya & Stirton3568	
2	[1] *O. beanianum* sp. nov*.* Muasya & Stirton3350	
3	[1] *O. bracteolatum limnophilum* sp. nov*.* Muasya & Stirton3204	
4	[1] *O. bracteolatum limnophilum* sp. nov. Stirton13155	[4] *O. hirtum* Muasya & Stirton3373
	[2] *O. fruticans* Muasya & Stirton3397	[5] *O. mundianum* Muasya & Stirton3885
	[3] *O. fruticans* Muasya & Stirton3480	[6] *O. parviflorum* Muasya & Stirton3542
5	[1] *O. bracteolatum* Muasya & Stirton3164	
	[2] *O. bracteolatum* Muasya & Stirton3879	
	[3] *O. bracteolatum* Muasya & Stirton3963	
6	[1] *O. candicans* Muasya & Stirton3563	
	[2] *O. schutteae* Muasya & Stirton3575	
7	[1] *O. candicans* Muasya & Stirton3911	
8	[1] *O. crewii* Muasya & Stirton3264	[4] *O. virgatum* Muasya & Stirton3395
	[2] *O. virgatum* Muasya & Stirton3163	[5] *O. virgatum* Muasya & Stirton3908
	[3] *O. virgatum* Muasya & Stirton3191	
9	[1] *O. hamatum* Muasya & Stirton3306	
	[2] *O. hamatum* Muasya & Stirton3310	
10	[1] *O. hirtum* Muasya & Stirton3190	[4] *O. hirtum* Muasya & Stirton3878
	[2] *O. hirtum* Muasya & Stirton3326	[5] *O. hirtum* Muasya & Stirton3991
	[3] *O. hirtum* Muasya & Stirton3372	
11	[1] *O. lucens* Muasya & Stirton3570	
12	[1] *O. prodiens* Muasya & Stirton3845	
	[2] *O. prodiens* Muasya & Stirton3854	
13	[1] *O. rubicundum* Muasya & Stirton5982	
14	[1] *O. spicatum* Muasya & Stirton3445	
15	[1] *O. stachyerum* Muasya & Stirton3791	
16	[1] *O. stachyerum* Muasya & Stirton3837	
17	[1] *O. uncinatum* Muasya & Stirton3175	
18	[1] *O. velutinum* Stirton13106	
19	[1] *P. aculeata* Muasya & Stirton3170	[4] *P. verrucosa* Muasya & Stirton3269
	[2] *P. oreopola* Muasya & Stirton4370	[5] *P. verrucosa* Muasya & Stirton3905
	[3] *P. plauta* Muasya & Stirton3611	
20	[1] *P. aculeata* Muasya & Stirton3183	[23] *P. oreophila* Muasya & Stirton3464
	[2] *P. aculeata* Muasya & Stirton3405	[24] *P. oreopola* Muasya & Stirton3271
	[3] *P. aculeata* Muasya & Stirton3550	[25] *P. oreopola* Muasya & Stirton4376
	[4] *P. affinis* Muasya & Stirton3868	[26] *P. pinnata* Muasya & Stirton3403
	[5] *P. affinis* Muasya & Stirton3903 2	[27] *P. pinnata* Muasya & Stirton3407
	[6] *P. aphylla* Muasya & Stirton3400	[28] *P. pinnata* Muasya & Stirton3547
	[7] *P. arida* Muasya & Stirton4098	[29] *P. pullata* Muasya & Stirton3903 1
	[8] *P. asarina* Muasya & Stirton3907	[30] *P. rhizotoma* Muasya & Stirton3659
	[9] *P. axillaris* Muasya & Stirton3848	[31] *P. rigidula* Muasya & Stirton3390
	[10] *P. axillaris* Muasya & Stirton5874	[32] *P. sordida* Muasya & Stirton3579
	[11] *P.* cf. *latifolia* Muasya & Stirton4028	[33] *P. sordida* Muasya & Stirton3580
	[12] *P. elegans* Muasya & Stirton3591	[34] *P. speciosa* Muasya & Stirton3458
	[13] *P. fleta* Muasya & Stirton3341	[35] *P. speciosa* Muasya & Stirton3607
	[14] *P. forbesii* Muasya & Stirton3578	[36] *P. speciosa* Muasya & Stirton3610
	[15] *P. forbesii* Muasya & Stirton3592	[37] *P. suaveolens* Muasya & Stirton4975
	[16] *P. gigantea* Muasya & Stirton3203	[38] *P. triflora* Muasya & Stirton3862
	[17] *P. imminens* Muasya & Stirton3596	[39] *P. usitata* Muasya & Stirton3194
	[18] *P. ivumba* Muasya & Stirton3374	[40] *P. usitata* Muasya & Stirton3440
	[19] *P. keetii* Muasya & Stirton3599	[41] *P. usitata* Muasya & Stirton3528
	[20] *P. laevigata* Muasya & Stirton3457	[42] *P. usitata* Muasya & Stirton3541
	[21] *P. latifolia* Muasya & Stirton3646	[43] *P. usitata* Muasya & Stirton4071
	[22] *P. odoratissima* Muasya & Stirton3557	[44] *P. verrucosa* Muasya & Stirton4371
21	[1] *P. alata* Muasya & Stirton3262	
	[2] *P. alata* Muasya & Stirton3398	
	[3] *P. alata* Muasya & Stirton3901	
22	[1] *P. alata* Muasya & Stirton3880	
	[2] *P. laxa* Muasya & Stirton3548	
	[3] *P. laxa* Muasya & Stirton3870	
23	[1] *P. arborea* Muasya & Stirton3212	[7] *P. glaucescens* Muasya & Stirton3289
	[2] *P. axillaris* Muasya & Stirton3827	[8] *P. ivumba* Muasya & Stirton3165
	[3] *P. axillaris* Muasya & Stirton3834	[9] *P. pinnata* Muasya & Stirton3169
	[4] *P. brilliantissima* Muasya & Stirton3621	[10] *P. pinnata* Muasya & Stirton3172
	[5] *P. congesta* Muasya & Stirton5462	[11] *P. repens* Muasya & Stirton3168
	[6] *P. filifolia* Muasya & Stirton4321	[12] *P. repens* Muasya & Stirton3809
24	[1] *P. arborea* Muasya & Stirton3248	[5] *P. odoratissima* Muasya & Stirton3532
	[2] *P. arida* Muasya & Stirton3526	[6] *P. pinnata* Muasya & Stirton3171
	[3] *P. asarina* Muasya & Stirton3476	[7] *P. usitata* Muasya & Stirton4344
	[4] *P. asarina* Muasya & Stirton3552	[8] *P. usitata* vigilans sp. nov*.* Muasya & Stirton4340
25	[1] *P. fleta* Muasya & Stirton3273	
26	[1] *P. fleta* Muasya & Stirton3342	[6] *P. imbricata* Muasya & Stirton3904
	[2] *P. imbricata* Muasya & Stirton3396	[7] *P. imbricata* Muasya & Stirton4030
	[3] *P. imbricata* Muasya & Stirton3399	[8] *P. verrucosa* Muasya & Stirton3353
	[4] *P. imbricata* Muasya & Stirton3439	[9] *P. verrucosa* Muasya & Stirton3357
	[5] *P. imbricata* Muasya & Stirton3544	
27	[1] *P. glaucescens* Muasya & Stirton3312	
28	[1] *P. laxa* Muasya & Stirton4325	
29	[1] *P. muirii* Muasya & Stirton4181	
30	[1] *P. oligophylla* Muasya & Stirton3798	
31	[1] *P. oreophila* Muasya & Stirton3463	
32	[1] *P. pinnata* Muasya & Stirton3186	
	[2] *P. pinnata* Muasya & Stirton3189	
33	[1] *P. pullata* Muasya & Stirton3178	
34	[1] *P. restioides* Muasya & Stirton3216	
	[2] *P. sparsa* Muasya & Stirton3567	
	[3] *P. speciosa* Muasya & Stirton3456	
35	[1] *P. usitata* ssp. nov*. usitata* Muasya & Stirton3414	
36	[1] *P. usitata* ssp. vigilans sp. nov Muasya & Stirton3415	
37	[1] *P. verrucosa* Muasya & Stirton3270	

**Figure 6.**
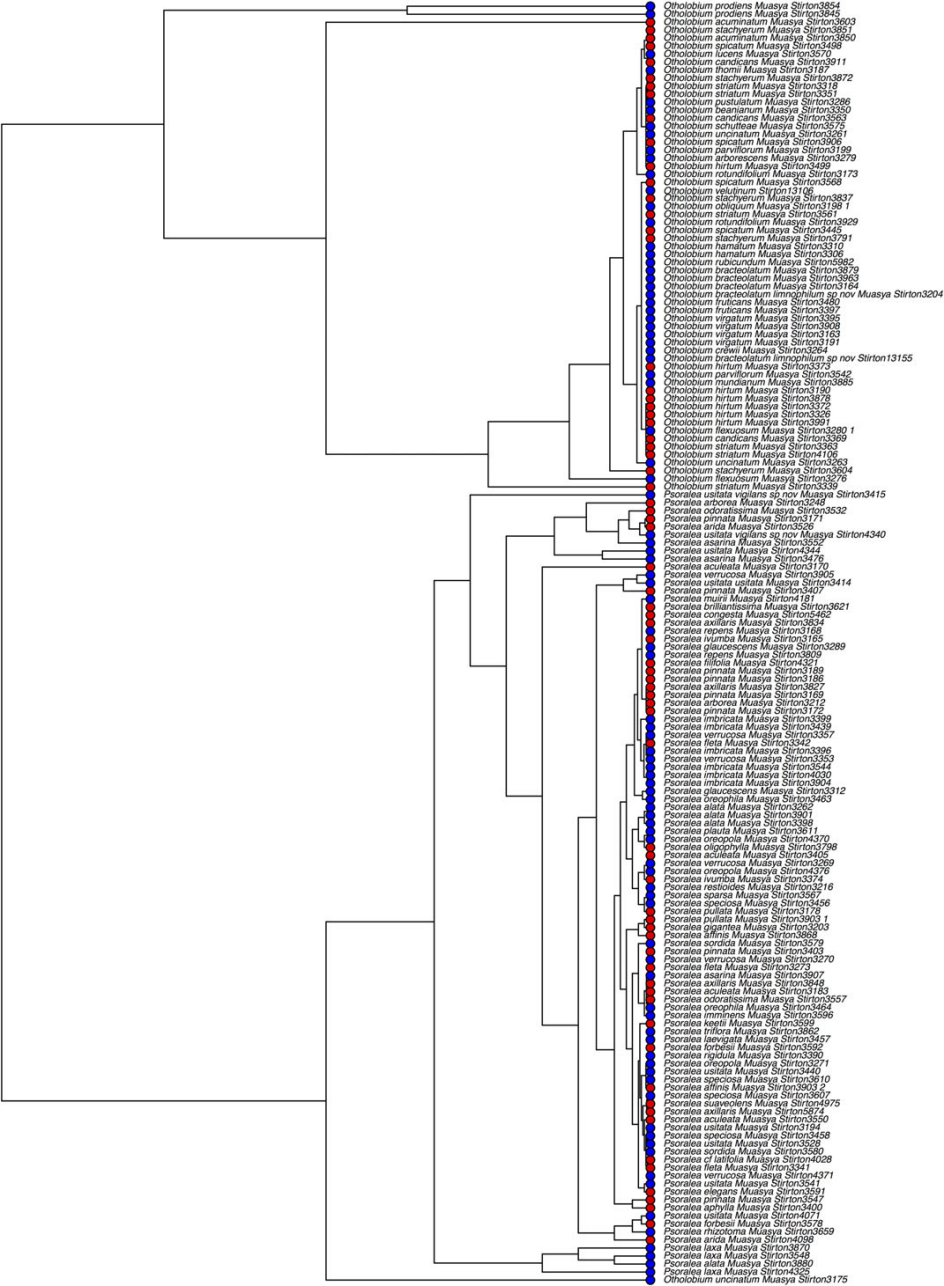
Maximum-likelihood tree of Psoraleoid legumes derived from a combination of the core DNA barcodes *matK* and *rbcLa* showing the distribution of fire-survival and regeneration strategies as reseeders (red) versus resprouters (blue).

Lastly, we found a weak but significant phylogenetic signal in the affinity of lineages to fire-survival and regeneration strategies. This was significant under the Brownian motion model (*D*_resprouters_ = 0.797, *P* = 0.003 and *D*_reseeders_ = 0.798, *P* = 0.002, where *D* = 0 corresponds to a Brownian motion model, and *D* = 1 indicates no phylogenetic signal) (Fig. 6). Multiple origin of reseeder habit is observed in both genera, but it is predominant in *Psoralea* (Fig. 6).

## Discussion

A key criterion for a standard plant barcode is universality, meaning that the DNA barcode should be easily recovered from all plants, ideally with a single primer pair (CBOL 2009). Our amplification and sequencing success was higher for *rbcLa* than for *matK*, consistent with the results of several other studies that sampled broadly across land plants (e.g. Lahaye *et al*. 2008; CBOL 2009; Xiang *et al*. 2011*a*; Saarela *et al*. 2013). Recovery of *rbcLa* was higher (98.1 %) than *matK* in this study. This corresponds to the results of other studies on plants in which *rbcLa* recovery ranged from 90 to 100 % (Fazekas *et al*. 2008; Lahaye *et al*. 2008; CBOL 2009; Jeanson *et al*. 2011; Pang *et al*. 2011; Xiang *et al*. 2011*a*; Kuzmina *et al*. 2012; Saarela *et al*. 2013).

Several other criteria have also been defined for the identification of the best DNA barcode marker (Hebert *et al*. 2004*a*; Kress and Erickson 2007; Lahaye *et al*. 2008; CBOL 2009). Firstly, it should exhibit a barcode gap, i.e. higher genetic variation between species than within species (Meyer and Paulay 2005). Secondly, it must provide a maximal discrimination among species. We measured the efficacy of the core plant DNA barcode regions (*matK* and *rbcLa*) (CBOL 2009) to identify African Psoraleoid legumes using the two approaches: ‘barcode gap’ and discriminatory potential (Meyer and Paulay 2005). We found that interspecific distance is significantly greater than intraspecific distance. Our mean distances correspond to the results obtained in other plant groups such as Myristicaceae (Newmaster *et al*. 2008), Rosaceae (Pang *et al*. 2011), *Taxus* L. (Taxaceae) (Liu *et al*. 2011) and in regional Canadian Arctic Flora (Saarela *et al*. 2013). The second approach was that of Meier *et al*. (2006), i.e. comparing the smallest interspecific versus the greatest intraspecific distances, instead of comparing the mean distances alone. This approach also reveals the existence of a barcode gap, thus confirming the barcode potential of all the candidates. However, the combination of *matK* and *rbcla* data sets (*matK* + *rbcla*) in all the cases showed greater intraspecific variation than the individual regions alone. This supports the recommendation of the CBOL (2009) that a combination of the two regions (*matK* and *rbcLa*) is the preferred standard barcode region for plants.

In addition, we found that all the three data sets have a strong discriminatory power (100 %) in identifying individuals to their respective genera within the Psoraleoid legumes using the near-neighbour and the best close match methods. This supports the utility of DNA barcoding as a means to identify and allocate species between the two genera. Multiple other studies have demonstrated that the core barcode loci routinely provide high discrimination at the genus level, usually >90 % (e.g. Kress *et al*. 2009; Saarela *et al*. 2013). Accordingly, we found that *rbcLa* and *matK* loci singly distinguish 100 % of genera in our data set. However, their application within species yielded a poor discrimination success, i.e. <50 % with more proportion of ambiguity (51 % *matK* + *rbcLa* data set to 79 % in *rbcLa* data set; Table 2). This result is not surprising, given that several other plant studies have reported poor utility of the core DNA barcodes at lower taxonomic level especially among closely related species and in taxa characterized by recent rapid radiation and hybridization. For example, Clement and Donoghue (2012) reported low levels of discrimination and genetic variation among closely related species of *Viburnum*. Similarly, Xiang *et al*. (2011*b*) reported that *rbcLa* alone was unable to distinguish genera within Juglandaceae, and neither *rbcLa* nor *matK* could discriminate species of *Berberis*, *Ficus* or *Gossypium* (Piredda *et al*. 2011). In taxa with hybridization issues, for example, *Quercus*, *matK* and *rbcLa* were unable to distinguish any of the 12 sympatric species examined (Roy *et al*. 2010). The possible causes of the poor discrimination of the species in Psoraleoid legumes observed in this study can be attributed to their recent rapid radiation (Egan and Crandall 2008) and multiple instances of strong hybridization (A. Bello, C.H. Stirton, S.B.M. Chimphango, A.M. Muasya, in preparation; see examples in paragraph below) among the species. Given these caveats, it is clear that additional variable loci are necessary to improve the within-species discrimination success as recommended by the CBOL (2009).

Another feature of interest is the low congruence in assigning morphologically delimited species to genetic species. Several reasons could account for this. Firstly, it could suggest that species are generally not monophyletic (Rieseberg and Brouillet 1994). Secondly, the mismatch could be due to poor performance of the DNA barcodes resulting in over-splitting of taxa. Thirdly, it could be that speciation events do not always match morphological differences, indicating that rapid changes in morphology can occur with minimal evolutionary change (Adams *et al*. 2002). Fourthly, it could indicate that taxa whose multiple accessions are appearing in diverse clades represent cryptic species, where broad morphological concepts on species are masking genetic patterns. This may be true in *Otholobium* where widespread species (*O. candicans*, *O. striatum* and *O. hirtum*) may be treated too broadly. Hybridization may account for some of the patterns in *Psoralea* as some of the taxa have been observed forming hybrids in the field, e.g. *P. pinnata* × *P. aculeata*, *P. sordida* × *P. forbesii* and *P. intonsa* × *P. oreopola*.

In general, there was a weak but significant phylogenetic signal in fire-survival and regeneration strategies of lineages as reseeders or resprouters in Psoraleoid legumes than would be expected by chance. Lineages show significant phylogenetic conservatism in their affinity to fire-survival and regeneration strategies with more clustering of resprouters at the tip of the phylogeny than might be expected by chance. Our phylogeny suggests a multiple origin of these traits implying that the species inherited the resprouting trait from their most recent common ancestor. We hypothesize that the scattering of the reseeding trait across the phylogenetic tree was the result of independent evolutionary events (convergent evolution), probably as a response to fire. It could also mean that the character was inherited from a more ‘basal’ ancestor of the group and then ‘switched off’ in some species but not in others again, in response to fire. However, this remains hypothetical at this stage, pending the availability of more data.

Legumes are regarded as one of the most successful families of flowering plants on Earth both from evolutionary and ecological perspectives, owing to their flexible adaptation to different environments (Rundel 1989). This is evident in the way resprouters and reseeders have evolved to survive in their respective microhabitats in the CFR (Schutte *et al*. 1995), and frequently dominant in after-fire landscapes. Previous comparative studies on these functional groups have focussed on aspects of taxonomy and physiology (Schutte *et al*. 1995; Power *et al*. 2011). Here, we provide evidence of a weak but significant phylogenetic signal in fire response trait of lineages as reseeders or resprouters in Psoraleoid legumes than expected by chance. Schutte *et al*. (1995) suggested that there is a substantial difference between resprouters and reseeders, adding that gene flow between resprouting parents and their offspring may occur over time, since the parents are not killed by fire. Seed set does occur in resprouters but is generally very poor and may not occur over a number of fire episodes. The seeds of resprouters are generally larger than those produced copiously by all reseeders (C. H. Stirton, pers. obs.).

In contrast, temporal isolation in gene flow might occur in reseeding taxa, as there is less chance of interbreeding between parents and offspring, and thus, each new generation may be a cohort of its own. It is not known how much seed remains in the seed bank and it is possible that some seeds may germinate in a later fire episode. It should be borne in mind, however, that parents and offspring could coexist if fires are patchy, if fire temperature affects the proportion of the seed bank that can be stimulated to germinate, if fires are too hot and if the seed bank comprises different genetic cohorts. The consequence of these is that speciation would more readily occur in reseeders, as interbreeding between parents and their progeny is unlikely. Given these caveats, our results provide some extrinsic support for the idea that reseeders speciate faster than resprouters as the number of reseeding species in our study outnumbered that of the resprouters. Schutte *et al*. (1995) reported that there is a faster rate of speciation and differentiation within reseeders, than in resprouters, but did not provide any genetic evidence for this. Most reseeding species of legumes in the CFR are short lived (ca. 8−15 years), with few exceptions, e.g. in *Podalyria calyptrata* and in some forest margin species of *Virgilia* with relatively long lifespans (>40 years). In the younger genus *Psoralea*, there are more reseeders than resprouters, whereas in the older genus *Otholobium*, there are more resprouters than reseeders and fewer species overall. Among the Psoraleoid legumes, reseeders are frequently observed on wet valleys near mountain streams, while resprouters are common in drier habitats, a phenomenon also observed in African Restionaceae, which shares increased diversification in reseeders (Litsios *et al*. 2014).

## Conclusions

This study showed that DNA barcoding may be useful in species identification and in inferring the impacts of recurrent fires on gene flow in resprouting and reseeding taxa in the CFR. In general, we showed that Psoraleoid legumes of the CFR exhibit a barcoding gap with high scores for correct identification of individuals to their respective genera. We found a considerable match between genetic and morphologically delimited species supporting the discriminatory power of DNA barcoding. We also found that lineages in Psoraleeae showed a weak but significant phylogenetic conservatism in their affinity for different fire response trait with more clustering of resprouters in *Psoralea* at the tip of the phylogeny than expected by chance. Our phylogeny suggests a convergent origin of the reseeding trait in African Psoraleoid genera. We conclude that these novel microevolutionary patterns might be acting continuously over time to produce multi-scale regularities of biodiversity especially in a biodiversity hotspot as the CFR.

## Accession Numbers

All data for the project were managed in the BOLD database in a project called ‘Fabaceae@UCT’ (project code FAUCT). Detailed voucher information, including the scientific names of taxa sampled, BOLD ID numbers, collectors and collection numbers, for all sequences are given in Table 1.

## Sources of Funding

This study was supported by grants from the South African National Research Foundation (NRF; A.M.M.); Nigeria Tertiary Education Trust Fund (NTETF)/Umaru Musa Yar'adua University Katsina, Nigeria (Fellowship Grant; A.B.) and University of Cape Town, J. W. Jagger Centenary Gift Scholarship (to A.B.).

## Contributions by the Authors

A.B. and B.H.D. performed the data analyses and were involved in writing and editing; C.H.S., A.M.M., S.B.M.C. and A.B. performed the fieldwork and were involved in writing and editing; M.v.d.B. and O.M. provided contribution to the concept and the design of the work and also handled the sequencing activities. All the authors read and approved the final manuscript.

## Conflict of Interest Statement

None declared.
